# Factors associated with reinfection of syphilis in reference centers for sexually transmitted infections

**DOI:** 10.1590/S1518-8787.2017051006432

**Published:** 2017-06-20

**Authors:** Valéria Correa de Almeida, Maria Rita Donalisio, Ricardo Cordeiro

**Affiliations:** I Faculdade de Medicina São Leopoldo Mandic.; Secretaria Municipal de Saúde de Campinas. Campinas, SP, Brasil; II Departamento de Saúde Coletiva. Faculdade de Ciências Médicas da Unicamp. Campinas, SP, Brasil

**Keywords:** Syphilis, epidemiology, Risk Factors, HIV Infections, Sexually Transmitted Infections, prevention & control

## Abstract

**OBJECTIVE:**

We aim to analyze trend of syphilis and factors associated with recurrent episodes of syphilis among adults and adolescents attended in a STI/AIDS reference centers in Campinas, state of São Paulo, 2004 to 2012.

**METHODS:**

Medical records, pharmacy data, and notification database were accessed to analyze trends of syphilis and sociodemographic, epidemiological and clinical variables associated with reinfection of syphilis. After univariate analysis, a hierarchical logistic regression model was adjusted to analyze variables associated with more than one episode of syphilis (dependent variable). First step (sex, age, and years of schooling) were tested and in the second, epidemiological and clinical variables.

**RESULTS:**

A total of 1,009 episodes of syphilis were identified among 860 adolescents and adults, 117 individuals (13.6%) presented with more than one episode of syphilis. Factors associated with more than one episode of syphilis were sex (male) (OR = 4.28; 95%CI 1.31–14.0), age (OR = 1.02; 95%CI 1.00–1.04), homosexual/bisexual orientation (OR = 2.29; 95%CI 1.22–4.32), HIV coinfection (OR = 3.54; 95%CI 2.22–5.63), and absence of STI symptoms at the time of syphilis diagnostic (OR = 1.70; 95%CI 1.03–2.80).

**CONCLUSIONS:**

The number of cases of syphilis and proportion in relation to STI increased in recent years in a specific population attended in a STI/AIDS reference centers in Campinas. Association with HIV, homosexual/bisexual orientation and the silent clinical characteristic of cases confirm the necessity to implement more aggressive strategies to prevent the occurrence of syphilis and other STI in specific populations with higher disease risk.

## INTRODUCTION

Sexually transmitted infections (STI) are one of the most common global health problems. The World Health Organization (WHO) estimated that 498.9 million new cases of STI occurred worldwide in 2008, including 10.6 million cases of syphilis^25^. Syphilis is a systemic disease caused by the spirochaete *Treponema pallidum* and is one of the oldest and most well-known diseases, for which curative treatment is available and efficient. Several studies have recently reported its resurgence, particularly in specific populations, especially in communities of men who have sex with men (MSM) in many parts of the world^6-8,12,23^.

Brazil has required compulsory notification for congenital and gestational syphilis since 1986 and 2005, respectively. However, the notification of syphilis in men and non-pregnant women became mandatory only in 2010. Therefore, few official data are currently available on this population in the country.

A low prevalence of syphilis in the general Brazilian population has been detected^15,18^. A hospital-based cohort study with 23,894 postpartum women has registered prevalence of 1.02%^5^. A national study within the Army has shown a prevalence of 0.55% in almost 36,000 individuals enlisted in 2007^7^. Prevalence of syphilis in pregnancy in Brazil was 1.6% in 2004^15^. Moreover, this disease appears to commonly affect specific populations. Prevalence among prisoners in a city of the state of São Paulo was 3.0% in 2003^4^, 19.7% among sex workers in the port cities of the State of Santa Catarina in 2009^22^, and 2.5 to 3.5% in a reference clinic for STI in Manaus from 2005 to 2008^16^.

Few studies have addressed recurrent syphilis in Brazil^4,15^. Data analyzed by the Centers for Disease Control and Prevention (CDC) showed that 20% of syphilis cases among MSM were due to reinfections in Baltimore, USA, between 2010 and 2011^3^. A study conducted in Denmark has shown that 14.8% of the population with syphilis had more than one episode during a five-year period^19^. Similarly, in San Francisco, USA, between 2001 and 2002, 6.7% of the individuals already diagnosed with syphilis presented a new primary infection within one year^17^, and a review describing cases of syphilis in California, USA, between 2002 and 2006, reported a reinfection rate of 5.9% in MSM^10^.

In this study, we have aimed to analyze the temporal distribution of syphilis among adults and adolescents attended in two reference centers for STI/AIDS and study the sociodemographic, clinical, and epidemiological variables associated with recurrence of syphilis, between 2004 and 2012.

## METHODS

This is a study on the infection and reinfection of syphilis in adults and adolescents of both genders treated in two reference centers for STI/AIDS of the Health Department of Campinas, State of São Paulo, Brazil (the Reference Center for STI/AIDS and the Testing and Counseling Center of Ouro Verde Hospital Complex) from January 2004 to December 2012. These reference centers cover approximately 70% of all HIV-infected individuals in the municipality of Campinas.

Data were collected from medical records, pharmacy, and the Information System of Sexually Transmitted Diseases (SINDST), using the name and record number to link patient information. Notification for STI was implemented in these centers in 2000. Variables used were sociodemographic (gender, age, years of schooling), epidemiological (route of transmission, sexual orientation, monogamous relationship in the last year, condom use and frequency, and previous STI), and clinical (coinfection of HIV, symptoms of STI, and clinical form of syphilis at the moment of diagnosis).

In this study, we included patients treated for syphilis, notified in the SINDST, and those who had a positive nontreponemal test result (Venereal Disease Research Laboratory test – VDRL) and a positive serology result for the treponemal test – *Treponema pallidum* hemagglutination assay (TPHA), Fluorescent Treponemal Antibody-Absorption test (FTA-ABS), or Chemiluminescence immunoassay (CLIA) for syphilis. Only the first episode of syphilis notified was considered to evaluate the time trend of disease. Syphilis testing is performed in all HIV patients and in all individuals with suspected infection. Awareness of the HIV status was identified in medical records.

Reinfection was considered when new cases of syphilis were diagnosed in patients who had previous diagnosis and completed treatment, verified in pharmacy and medical records, and had a positive nontreponemal test after 10 weeks of the last treatment, according to the protocol of the Brazilian Ministry of Health^15^. Cure was considered when the decline in VDRL levels was at least two titers in the period of one year. All patients receiving care in the reference centers were treated according to the recommendations of the Ministry of Health of Brazil and the CDC^3,15^.

We analyzed the temporal trend of cases according to the year of report and the proportion of infections of syphilis in relation to other STI. As we do not have the population of reference from which cases came from, we used the annual ratio of syphilis/total of STI.

We analyzed the trends of annual distribution of individuals with more than one episode of syphilis according to the year of notification of the first episode. Based on the functional relationship observed in the annual trend curve of reinfection, a linear regression was adjusted and the correlation coefficient (r^2^) was calculated. We needed to transform the variable ‘year’ into a year-centralized variable (year minus the midpoint of the series), since, in linear regression models of time trends, the points of the series are often self-correlated.

After univariate analysis, a hierarchical multiple logistic regression model was adjusted in two stages, with the presence of multiple episodes of syphilis as the dependent variable, and the sociodemographic, epidemiological, and clinical variables as the independent ones. On the higher level, we added the contextual variables and, on the lower level, we added the individual covariates. The multiple logistic regression followed the hierarchical model: in the first stage, we included the sociodemographic variables (age, gender, and years of schooling). In the second stage, we added to the first set the epidemiological and clinical variables that had significance in the adjustment for confounding factors from the same hierarchical level. Therefore, we included in the model the variables that showed significant association with more than one episode of syphilis in the simple analysis with p < 0.20, and only those that showed a value of p < 0.05 remained in the final model. Odds ratios (OR) and 95% confidence intervals (95%CI) were estimated.

The study was approved by the Research Ethics Committee of the Faculdade de Ciências Médicas of Universidade Estadual de Campinas, State of São Paulo, Brazil (Protocol 510370).

## RESULTS

The profile of individuals with syphilis between 2004 and 2012 showed that 3,106 cases of STI were reported to the SINDST database and treated in reference centers for STI and HIV/AIDS in Campinas. Syphilis accounted for 1,009 (32.5%) of all reported and treated episodes of STI diagnosed in 860 individuals.

**Figure 1:**
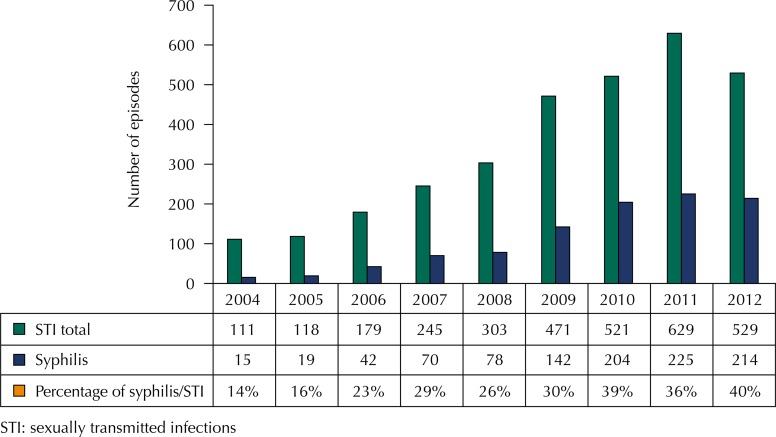
Total number of STI, syphilis, and proportion of syphilis in relation to total STI and year of notification. Campinas, State of São Paulo, Brazil, 2004 to 2012.

Figure 1 presents the temporal trend of infections of syphilis and the percentage of syphilis in relation to all STI cases. Among the 860 individuals who presented at least one episode of syphilis, 117 individuals (13.6%) were diagnosed with more than one.

STI: sexually transmitted infections

**Figure 2:**
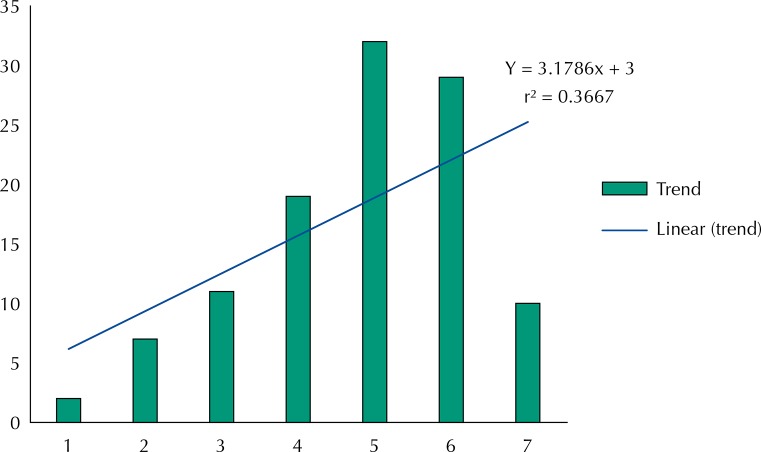
Annual distribution of individuals with recurrence of syphilis according to the year of notification of the first episode. Campinas, State of São Paulo, Brazil, 2004 to 2012.

Figure 2 shows the distribution of the 117 individuals who presented more than one episode of syphilis according to the year in which the episode was first notified. A linear trend shows correlation coefficient (r^2^) of 0.3667. A significant difference in the mean infection rate between the time periods analyzed was observed (p ≤ 0.05), with most individuals being infected for the first time in the last 4 years of the study.

Most participants were male (90.8%), had a high level of education (71.8% studied for more than 11 years), and self-reported as homosexual or bisexual, i.e., MSM (57.4%). Data regarding sexual partners were obtained from 849 patients, and 458 (53.9%) declared not to be in monogamous relationships. Although it is not a reliable information, 76.2% individuals reported condom use, and only 78 (9.1%) reported regular use. Approximately 102 (11.9%) individuals suffered from previous STI other than infections of HIV or syphilis (gonorrhea, chancroid, human papillomavirus (HPV), molluscum contagiosum). At the moment of diagnosis of syphilis, 596 (69.3%) individuals did not complain of any symptoms (ulcers on the skin or mucous membrane, rash or lymphadenopathy, signs and symptoms of tertiary syphilis), suggesting that latent syphilis was the predominant clinical form, as reported in medical records of 606 cases (70.5%). Among the 117 patients who had more than one episode of syphilis, 23 (2.7%) presented three or more episodes.

Coinfection with HIV was present in 377 (43.8%) individuals, and 86 (22.8%) had more than one episode of syphilis, among which 57 (66.3%) were diagnosed with HIV infection before developing syphilis and 29 (33.7%) became aware of their HIV-positive status at the diagnosis of syphilis.

Table 1 show the variables associated with more than one episode of syphilis in a univariate analysis. The final results of the hierarchical regression analysis are presented in Table 2. A significantly higher prevalence of males, HIV coinfection, and non-monogamous relationship was observed among the individuals presenting with more than one episode of syphilis. The absence of symptoms at the moment of diagnosis of syphilis was positively associated with presenting more than one episode of syphilis (Table 2).

**Table 1:** Epidemiological and clinical variables associated with recurrence of syphilis using a univariate analysis, in two reference centers for STI. Campinas, State of São Paulo, Brazil, 2004 to 2012.

Variable	More than one episode of syphilis		One episode of syphilis		_crude_OR	95%CI
	
	n	%	n	%		
Gender						
Male	114	97.4	667	89.8	**4.33**	**1.34–13.96**
Female	3	2.6	76	10.2	1	
Age (years)						
Mean	34.4		32.5		1.02	0.99–1.03
Median	31		31			
Standard deviation	10.6		10.5			
Schooling (years)^a^						
0–4	9	7.8	46	6.2	0.984	0.44–2.198
5–11	73	62.9	524	70.6	0,701	0.45–1.09
≥ 12	34	29.3	172	23.2	1	
Sexual identity						
Homo/Bisexual	84	71.8	482	64.9	1.06	0.62–1.83
Heterosexual	13	11.1	141	19.0	0.56	0.27–1.19
Unknown	20	17.1	120	16.1	1	
Monogamous relationship^b^						
Yes	43	36.8	348	47.4	0.66	0.44–0.99
No	72	61.5	386	52.6	1	
Number of partners/year						
Mean	10.7		39.1		0.99	0.98–1.01
Median	10		5			
Standard deviation	30.3		130.1			
Regular use of condom						
Yes	98	83.8	549	74.9	0.94	0.69–1.49
Never/Sometimes	19	16.2	184	25.1	1	
HIV infection						
Yes	86	73.5	291	39.2	**4.31**	**2.78–6.67**
No	31	26.5	452	60.8	1	
Previous STI^c^						
Yes	14	12.0	88	11.8	1.01	0.55–1.83
No	103	88.0	655	88.2	1	
Symptoms of STI^d^						
Yes	24	20.5		32.3	1	
No	93	79.5	503	67.7	**1.84**	**1.15–2.97**
Clinical form of syphilis						
Primary						
Yes	7	6.0	116	15.6	0.34	0.16–0.76
No	110	94	627	84.4	1	
Secondary						
Yes	17	14.5	110	14.8	0.98	0.56–1.70
No	100	85.5	633	85.2	1	
Latent						
Yes	91	77.8	515	69.3	1.55	0.97–2.46
No	26	22.2	228	30.7	1	
Neurosyphilis						
Yes	2	1.7	2	0.3	6.44	0.89–46.19
No	115	98.3	741	99.7	1	
Total	**117**	**13.6**	**743**	**86.4**		

STI: sexually transmitted infections

Unknown: (^a^) 2; (^b^) 11.

^c^ History of previous STI other than HIV or syphilis – gonorrhea, chancroid, human papillomavirus (HPV), molluscum contagiosum.

^d^ Symptoms of STI at the moment of syphilis diagnosis.

Statistically significant associations are presented in bold.

**Table 2:** Variables associated with recurrence of syphilis using a hierarchical logistic regression analysis in two reference centers for STI. Campinas, State of São Paulo, Brazil, 2004 to 2012.

Variable	First step		Second step	
	
	_adj_OR^a^	95%CI	_adj_OR^b^	95%CI
Age	1.02	1.00–1.04		
Gender (male)	4.28	1.31–14.00		
HIV/AIDS			3.54	2.22–5.63
MSM^c^			2.29	1.22–4.32
No symptoms of STI^d^			1.70	1.03–2.80

STI: sexually transmitted infections; MSM: men who have sex with men

^a^ Adjusted odds ratio for sociodemographic variables.

^b^ Adjusted odds ratio for sociodemographic, epidemiological, and clinical variables.

^c^ The variable “men who have sex with men” included homosexual and bisexual orientation.

^d^ No symptoms of STI at the moment of syphilis diagnosis.

## DISCUSSION

An increase in the absolute numbers of syphilis and in the proportion to other STI was observed in the users of reference centers in Campinas. Although the analyzed population does not represent all adolescents and adults of Campinas, this study suggests that cases of syphilis significantly increased in this specific population during the study period.

These findings suggest an expansion of the transmission of *Treponema pallidum* in recent years in the specific population attended by reference centers for STI in Campinas. Although this is not a population based study, those are traditional and stable reference health services with good coverage, resulting in an approximation of the real trend and reliable estimators of the disease in the city. Indeed, health services did not experience important changes in the organization, such as changes in the medical routine, physical structure, expansion of medical consultations, or specific preventive campaigns to justify the increase in cases and the demand for reference centers. The information system on dispensing drugs, medical records, and case reporting was implemented in the 90s, and it is of good quality.

Among the 860 patients with syphilis, 117 (13.6%) individuals presented recurrent infection during the study period. Data in the literature on reinfection of syphilis are limited. However, the results from Campinas are similar to those reported in Denmark^19^.

A higher notification of more than one episode of syphilis was observed particularly among MSM in this study, as shown by other authors^2,10,17^. Several hypotheses have been formulated for this phenomenon. Some studies have called attention to recent changes in the sexual behavior of this specific population, and speculate that this is due to the understanding that HIV infection is now considered a chronic, treatable disease. This notion has led to a reduction in the preventive measures adopted for STI^10^. Some authors have also suggested that oral sex (sometimes preferred in order to diminish the risk of HIV transmission), when associated with the increased use of recreational drugs such as methamphetamines, is an important route for syphilis transmission^2^. The use of prescription drugs, such as sildenafil citrate^20^, for recreational purposes and to facilitate the establishment of sexual encounters – for example, over the internet – are also a contributing factor for new sexual behaviors, especially among MSM^14^. Thus, similar to HIV, we can suspect that the epidemic of syphilis is concentrated on specific populations, as some groups appear to be more affected than the general population in Brazil.

An association between recurrent syphilis and HIV coinfection has also been reported by Phipps et al.^17^, who have found that the probability of HIV-positive individuals to present more than one episode of syphilis was almost five times higher than for HIV-negative individuals. Numerous factors contribute to this finding: syphilis and HIV infections share the same form of transmission, and similarly to syphilis, the prevalence of HIV infection in Brazil is higher in specific populations, such as sex workers and MSM, with a reported prevalence of 4.9% and 10.5%, respectively^15^. In our study, among the co-infected individuals who presented with more than one episode of syphilis (n = 86), one-third (n = 29) were diagnosed with HIV simultaneously with their diagnosis of syphilis, whereas two-thirds (n = 57) were already aware of their HIV-positive status and had developed syphilis subsequently.

The routine screening for syphilis among HIV-positive patients was implemented in 2004. It is worth noting that HIV-infected individuals receive continuous clinical follow-ups, including annual screenings for syphilis. This consequently increases the probability of a diagnosis of syphilis, compared to individuals who are HIV-negative or unaware of their HIV status, who may have been infected more than once, without being diagnosed^1^. In part, it can explain the association between HIV and reinfection of syphilis.

Another factor that has been described as a facilitator for the transmission of syphilis among HIV-infected individuals is known as serosorting^9^, that is, HIV-positive individuals seeking sexual partners with the same serological condition, to avoid the use of condoms and to maintain more stable relationships. However, this facilitates the transmission of syphilis and other STI. Several studies have noted that the increased number of syphilis cases in HIV-positive individuals is associated with the use of highly active antiretroviral therapy (HAART), as it is known that HIV-infected patients with undetectable viral load have a decreased probability of viral transmission. This diminishes the perceived importance of the preventive measures against HIV transmission, thus increasing the risk of other STI^11,21,24^.

The absence of symptoms, as well as the prevalence of the latent form of syphilis, demonstrates the silent behavior of the disease. It also suggests that individuals with more than one episode of syphilis generally do not display symptoms of other STI – a notion that contradicts the current clinical knowledge about the occurrence of STI. We hypothesize that this is either because the prevalence of syphilis is higher in the general population than previously thought, or because when individuals present with STI symptoms, intervention is usually aimed at treating the symptoms of the disease only (e.g., secretions, ulcers, or warts), and serology for syphilis is seldom tested despite the current recommendations^22^.

Limitations of this study include the use of secondary data sources and being restricted to a specific population treated in two reference centers for STI. Another limitation is a possible inaccuracy of the estimate of the first episode of syphilis and also reinfection confirmed with serology. Although this is a study of the users of a specific service with good coverage, there was no certainty about the number of cases of reinfection that may have not returned to the center. These losses could affect the association estimates; however, our results may be sentinel, that is, a marker of the tendency of reinfection in the community attended by the health service. Findings of reinfection of syphilis in individuals with HIV may be partly explained by an improvement in diagnoses and notification methods and a higher risk perception of patients related to counseling that lead them to consult when they suspect reinfection.

In Brazil, the efforts to control syphilis are largely focused on congenital syphilis, although the disease in men and non-pregnant women should be better monitored as well, as each infected pregnant woman is likely associated with a syphilis-infected partner. The compulsory notification of acquired syphilis, introduced in 2010, may contribute to an improved understanding of the behavior of the infection within the population, even when facing the underreporting of cases. The data obtained in this study, similarly to previous findings in the literature, expose a higher prevalence of infection of syphilis in specific populations, and therefore, continuous interventions using public awareness campaigns are needed to address the issue.

Lastly, we have to question whether the recent Brazilian recommendations to reinforce and increase of antiretroviral treatment for HIV patients in order to prevent HIV transmission^13^ will contribute to an increase in the prevalence of other STI, especially if prevention campaigns do not incorporate more aggressively the prevention of other STI.
